# Juvenile idiopathic inflammatory myopathies assessment by magnetic resonance imaging: a scoping review of protocols, scoring systems, and applications

**DOI:** 10.1186/s42358-025-00494-z

**Published:** 2025-11-21

**Authors:** Vitor Tavares Paula, Clarissa Harumi Omori, Samuel Katsuyuki Shinjo, Daniel Brito de Araújo, Jessica Day, Adam Schiffenbauer, Claudia Saad Magalhães, Edoardo Conticini, Edoardo Marrani, Julio Brandão Guimarães, Lisa G. Rider, Mickael Essouma, Simone Appenzeller, Andrea Schwarz Doria, Adriana Maluf Elias

**Affiliations:** 1 Environmental Autoimmunity Group, Clinical Research Branch, National Institute of Environmental Health Sciences, National Institutes of Health, Bethesda, MD USA; 2 Department of Diagnostic & Interventional Radiology, Research Institute, The Hospital for Sick Children, University of Toronto, Toronto, ON Canada; 3 Pediatric Rheumatology Unit, Children’s Institute, Hospital Das Clínicas HCFMUSP, Faculdade de Medicina, Universidade de São Paulo, São Paulo, SP Brazil; 4 Pediatric Rheumatology Unit, Department of Pathology, Botucatu Medical School, São Paulo State University, Botucatu, São Paulo Brazil; 5 Internal Medicine Department, Universidade Federal de Pelotas, Pelotas, Rio Grande do Sul, RS Brazil; 6 Rheumatology Unit, Department of Medicine, Surgery and Neurosciences, University of Siena, Siena, Italy; 7 Rheumatology Unit, ERN-ReCONNET Center, Meyer Children’s Hospital IRCCS, Florence, Italy; 8 The Walter and Eliza Hall Institute of Medical Research, Melbourne, Victoria, Australia; 9 Royal Melbourne Hospital, Melbourne, Victoria, Australia; 10 The University of Melbourne, Melbourne, Victoria, Australia; 11 Universidade Federal de São Paulo, EPM-UNIFESP, São Paulo, Brazil; 12 Grupo Fleury Medicina E Saúde, São Paulo, Brazil; 13 Network of Immunity in Infection, Malignancy and Autoimmunity, Universal Scientific Education and Research Network, Yaounde, Cameroon; 14 Division of Rheumatology, Faculdade de Medicina FMUSP, Universidade de São Paulo, São Paulo, Brazil; 15 Department of Orthopedics, Rheumatology and Traumatology, University of Campinas, Campinas, SP Brazil; 16 Radiology Department, Children’s Institute, Hospital das Clínicas HCFMUSP, Faculdade de Medicina, Universidade de São Paulo, São Paulo, SP Brazil; 17 Department of Imaging, Hospital Israelita Albert Einstein, São Paulo, SP Brazil

**Keywords:** Idiopathic inflammatory myopathy, Juvenile dermatomyositis, Magnetic resonance imaging, Myositis, Scoping review

## Abstract

**Background:**

Our review assessed whole body (WB) and dedicated body-part magnetic resonance imaging (DedMRI) techniques, protocols, and inflammatory scoring systems, focusing on their clinimetric properties (reliability, validity, responsiveness) in clinical and research settings of patients with juvenile idiopathic inflammatory myopathies (JIIM).

**Methods:**

A comprehensive search of MEDLINE, EMBASE, and Cochrane databases from 2000 to 2024 identified relevant studies.

**Results:**

Sixteen studies enrolling JIIM patients with MRI were reviewed, which showed heterogeneity in objectives, methodologies, and scoring systems. Four (25%) studies used quantitative assessments, while 12 (75%) employed semi-quantitative or qualitative methods in scoring MRI. WB-MRI was performed in 3 (18.7%) studies, and DedMRI in 13 (81.2%). Muscle evaluation included assessments of edema [14 (87.5%) studies], fatty infiltration [4 (25%) studies], and atrophy [6 (37.5%) studies]. T1 images were used in 9 (56.2%) studies for measuring chronic changes, with coronal views reported in 6 (37.5%). Fluid-sensitive sequences (T2 with fat saturation, STIR) were employed in all studies and were obtained in the coronal plane in 9 (56.2%). These sequences were crucial for detecting soft tissue edema related to acute/subacute inflammation. One (6%) study included diffusion and T1 post-contrast sequences.

**Conclusion:**

There is significant heterogeneity in MRI protocols for evaluating JIIM. Standardized WB-MRI protocols are needed to ensure consistency and comparability across studies and institutions, optimize assessments of disease activity, treatment response, and follow-up in JIIM patients. Standardization should enhance the reliability of MRI for diagnosing and monitoring JIIM in clinical and research settings.

## Key-points

- Heterogenous qualitative, semi-quantitative, quantitative analysis and magnetic resonance imaging (MRI) protocols used to evaluate idiopathic inflammatory myopathies (IIM) in pediatric patients were observed.

- Subsequent research efforts should concentrate on establishing uniform protocols and scoring criteria to improve the reproducibility and accuracy of MRI evaluations in pediatric IIM.

## Introduction

The juvenile idiopathic inflammatory myopathies (JIIM) are a heterogeneous group of disorders characterized by chronic damage of the muscle due to a persistent immune-mediated process. In particular, juvenile dermatomyositis (JDM) is the most common type of JIIM. JDM is a systemic rheumatic autoimmune disease characterized clinically by muscle weakness and vasculopathy. The diagnosis and classification of JDM is based on Bohan and Peter’s criteria [1], and the European League Against Rheumatism/American College of Rheumatology (EULAR/ACR) criteria [2], which include muscle biopsy to assess for myopathic changes.

While the prognosis of JDM has improved with advancements in treatment, ongoing challenges remain, including disease flares, chronic muscle inflammation, muscle damage, and secondary morbidity due to medication and physical inactivity [3–5]. Owing to the invasive nature of electromyography and muscle biopsy [6–10] and their limitations in accurately defining disease activity and damage, magnetic resonance imaging (MRI) has been increasingly used for both diagnosis and follow-up of patients with JDM in clinical practice. However, its value still requires further clarification, especially regarding its application for diagnosis, monitoring and assessment of inflammatory muscular activity. MRI provides an accurate and reproducible assessment of the musculoskeletal system, being non-invasive, radiation-free and not dependent on the operator, making it particularly well-suited for young patients [11].

Whole body MRI (WB-MRI)-focused Myopathies Working Group within the International Myositis Assessment and Clinical Studies Group (IMACS) developed a protocol for a scoping review of the literature to determine the role of MRI in the diagnosis and assessment of JIIM and to evaluate its utility as an outcome measurement for pediatric patients with muscle inflammation and damage [12]. This manuscript builds upon the one previously published protocol by our group, presenting the results of the scoping review. In contrast to the protocol article, which described the methodology planned, the present study synthesizes the current evidence regarding MRI protocols, scoring systems, and applications in JIIM. Here, we report the results of this scoping literature review, focusing on MRI protocols, scoring systems, and clinical applications on patients with JIIM -particularly those with JDM. These findings are expected to contribute to future development of consensus recommendations for a standardized MRI assessment scale for pediatric patients with suspected JIIM.

## Materials and methods

Articles of interest were identified from January 2000 to December 2024 through searches on MEDLINE (via PubMed), EMBASE, and the Cochrane Library. The search strategy was structured according to the main concepts of the research question, namely the population (juvenile idiopathic inflammatory myopathies, juvenile dermatomyositis, pediatrics) and the assessment method (magnetic resonance imaging). Both keywords and controlled vocabulary terms (MeSH in PubMed and Emtree in EMBASE) were used. Filters were applied to restrict the results to studies involving humans, pediatric population, and publication years from 2000 to 2024. Equivalent search strategies were adapted for EMBASE and Cochrane Library. In addition, electronic search was complemented by a manual review of the reference lists of relevant articles [12].

Inclusion criteria were publications of JIIM in patients younger than 18 years of age diagnosed by the Bohan and Peter criteria [1] or the EULAR/ACR classification criteria for JIIM [2], with WB-MRI or dedicated body part MRI (Ded-BP-MRI) as the main assessment or as part of the study procedures and assessments.

The exclusion criteria were case reports or case series with fewer than five cases, review articles, conference abstracts and posters, opinion papers, commentaries, publications with no data concerning MRI techniques and their analysis, decision-analytic models, economic evaluations, or articles addressing etiopathogenic mechanisms of myopathies beyond inflammation (e.g., neuromyopathic etiopathogenesis). As the objective was to develop a standardized MRI scoring system, publications prior to the year 2000 were excluded, as imaging quality, MRI scanners generation, and acquisition techniques reported in earlier studies were not comparable to those of more recent investigations [12]. Publications including both juvenile and adult IIM patients were also excluded, since the focus of this review was to capture their specific clinical characteristics. Studies in which raw data or additional information were unavailable even after contacting the authors were also excluded, as were duplicate, salami-sliced, or plagiarized publications [12].

Each title and abstract were reviewed by two independent investigators, and then discussed among the working group. The investigators were then paired to complete the standardized data extraction and worksheets using the selected full manuscripts. Two experienced imaging experts (V.T.P and J.B.G) analyzed the MRI-reported methodology for all articles.

The information from the primary studies was descriptively synthesized according to the objectives of this scoping review. Data were independently extracted by two reviewers using a standardized template and subsequently organized into predefined categories relevant to the research question: clinical indications for MRI in patients with JIIM; MRI techniques and protocols; use of MRI scoring systems and approaches to data interpretation; comparisons between MRI findings and clinical and laboratory measures of disease activity, remission, flares, or damage; and the diagnostic performance of MRI in addressing the objectives proposed. The extracted data were summarized and systematically tabulated (Tables 1, 2 and 3) to enable a structured comparison of findings across studies.

**Table 1:** Study characteristics of magnetic resonance imaging in patients with juvenile idiopathic inflammatory myopathies from the scoping literature review

Authors [Ref]	Year	Country	Study design	JIIM (n)	Age	Disease duration	Current treatment	Female (%)	JIIM descriptors and activity measures
Hilário et al. [13]	2000	Brazil	Cross sectional	13 JDM	5.6–16.6 yrs (mean 11.7)	1.5–13.6 yrs (mean 4.5)	GC dose:0.59 (0-.2 mg/kg/d)	69.2	Muscle weakness: 68%; elevated muscle enzymes: 46% (elevated CK:83%); disease activity: 46%
Kimball et al. [14]	2000	USA	Prospective	26 JDM	3.7–17.7 yrs (mean 10.8)	1–82 mo (mean 31.7)	GC: 84.6% HCQ: 26.9% MTX: 42.3% Aza: 7.7% IVIG: 23%	73.0	Median (25%, 75%) Global disease activity score 2.0 (1.0, 3.0) Global skin disease activity score 2.0 (1.0, 2.0) Lower extremity proximal MMTscore 64.5(52.5, 72.0) CMAS extended score 44.5 (32.0, 46.0) CK level 0.1 (0.1, 0.4) Aldolase level 1.2 (0.9, 1.6) LDH level 1.0 (0.8, 1.2)
Huber et al. [15]	2004	USA	Prospective	98 JDM; 6 JPM; 4 overlap myositis (36 with MRI)	3.1–18.8 yrs (median 9.0)	0–132 mo (median 24.0)	NR	NR	Median values: CMAS: 44; CHAQ: 0.25; total MMT: 90.3; PGA of disease activity: 2.1; PGA of disease damage: 0.5; CK: 0.3; ALT: 0.5; AST: 0.9; LDH: 1.0; aldolase: 0.9
Maillard et al. [16]	2004	UK	Cross sectional	20 JDM	10 active JDM 3.8–11.8 yrs (mean 6.6) 10 inactive JDM 2.7–13.3 yrs (mean 6.9)	active JDM 0.3–6.7 yrs (mean 2.2) inactive JDM 1.3–8.0 yrs (mean 3.3)	NR	NR	Active JDM: PGA: 4.52; CMAS: 36; CHAQ: 1.71; manual neck strength: 2.9; myometry of the neck: 10.1 Inactive JDM: PGA:1.1; CMAS: 44; CHAQ: 0.712; manual neck strength: 4.8; myometry of neck 28.9
Maillard et al. [17]	2005	UK	Cross sectional	20 JDM	10 active JDM 3.8–11.8 yrs (mean 6.6) inactive 10 JDM 2.7–13.3 yrs (mean 6.9)	active JDM 0.3–6.7 yrs (mean 2.2) inactive JDM 1.3–8.0 yrs (mean 3.3)	NR	NR	Active JDM: PGA: 4.52; myometry of the neck: 10.1; baseline CK: 196; baseline LDH: 1137 Inactive JDM: PGA: 1.1; myometry of the neck: 28.9; baseline CK: 261 baseline LDH: 845
Sanner et al. [18]	2010	Norway	Cross sectional	59 JDM	mean: 9.1 (±4.5)	mean:16.9 (±10.6)		61	Median values; MMT: 78; CMAS: 50; SF36 PCS score: 53.9 24 (41%): positive ANA; 2 anti-SRP and anti-Jo1; 1 anti-Ku; 2 anti-Ro 52
Ladd et al. [19]	2011	USA	Retrospective	45 JDM	1–18 yrs (median: 6)	1 week-6 yrs (median: 4.5)	No GC before MRI	68.8	49%: limited disease; 51%: chronic disease
Davis et al. [20]	2011	UK	Retrospective	43 JDM	study: 18 mo-15 yrs diagnosis: mean: 6 yrs 11 mo	NR	NR	76.7	NR
Malattia et al. [21]	2014	Italy	Cross sectional	41 JDM	7.1–12.9 yrs (median: 8.8)	0.3–3.7 yrs (median:0.8)	Untreated: 9 GC:7 GC+MTX:14 GC+CP:14 HCQ: 15 IVIG: 5	53.6	Median values: MMT: 69; CMAS: 36; CHAQ: 0.9; PGA disease activity: 5.5; PGA overall damage: 0.0; DAS total: 10.0; MDI total:1.0; CPK:124; LDH: 607; AST: 37; CRP: 0.45; ESR: 14.0
Thyoka et al. [22]	2018	UK	Cross sectional	20 JDM	NR	NR	NR	NR	NR
Sakurai et al. [23]	2018	Japan	Retrospective	18 JDM	12 m-14 yrs (median:4.8)	before 2 mo (7 early-diagnosed JDM) after 2 mo (11 late-diagnosed JDM)		77.7	Muscle pain: 6 early-diagnosed, and 5 late-diagnosed 3 anti-MDA5; 6 anti-TIF-1γ; 100% aldolase elevated, 44% CK elevated
Mamyrova et al. [24]	2018	USA	Case-control prospective cross sectional study	21 JDM	median 12.0 yrs (9.2–15.6)	2.4 yrs (2.0–3.7)	GC (mg/kg/d) 0.29 [0.19–0.44] MTX and/or IV GC: 80% IVIG and/or HCQ: 70% CP: 20% AZA 15% TAC or CYC: 10% IFX: 5%	66.7	Median values: PGA VAS: 3.1; PGD VAS: 1.9; MMT proximal: 150; QMT knee extension maximum: 21.8; CMAS: 47; C-HAQ: 0.4; MDAAT: 5.5; CK: 31; LDH: 222; aldolase: 4.5
Sreelal et al. [25]	2021	India	Prospective	21 JDM; 9 Overlap	2–18 yrs (8.2 ± 3.9)			53.3	Proximal weakness lower limb: 3 (73.3%), 4 (16.7%), 5 (10%) AST mean: 89.8; ALT mean: 62.3; CK mean: 10.9; LDH mean: 576
Hu et al. [26]	2022	China	Retrospective	55 training cohort; 20 validation cohort	training cohort: mean: 6.9 ± 3.3 yrs validation cohort: mean: 7.7 ± 4.7 yrs			training cohort: 45.4 validation cohort: 45.0	CMAS mean scores: training cohort: 43.3; validation cohort: 43.4 ESR median values: training cohort: 9.0; validation cohort: 13.5 CRP median values: training cohort: 0.5; validation cohort: 0.7 CK median values: training cohort: 2.61; validation cohort: 2.50 EMG 2 myogenic damage: training cohort: 89.9%; validation cohort: 95% Myositis antibody positive: training cohort: 38.2%; validation cohort: 30% Anti-NXP2 antibodies: training cohort: 9.1%; validation cohort: 10% Anti-MDA5 antibodies: training cohort 9.1%; validation cohort: 15% Biopsy positive: training cohort: 94.6%; validation cohort: 94.1%
Yi et al. [27]	2024	USA	Retrospective	14 calcinosis 29 no-calcinosis	calcinosis: 4 yrs IQR (3–8) no-calcinosis: 7 yrs IQR (4–12)	calcinosis: 4 mo IQR (2–11.5) no-calcinosis: 2 mo IQR (1–4.5)		57 calcinosis 59 no-calcinosis	Median values: CK: calcinosis: 467; no-calcinosis: 4246 Aldolase: calcinosis: 12.2; no-calcinosis: 17.4 LDH: calcinosis: 1751; no-calcinosis: 2037 AST: calcinosis: 152; no-calcinosis: 243 ALT: calcinosis: 80; no-calcinosis: 160
Castro et al. [28]	2014	Brazil	Cross sectional	32 JDM 2 JPM	median: 12.2 yrs (5.5–18.9)	median: 4.2 yrs (0.19–13.9)	GC: 38.2% MTX: 76.4% HCQ:11.7% CP:11.7% Aza:2.9%	70	Median values: ESR: 9; CRP: 1.78; AST: 23; ALT: 17; aldolase: 6.25; LDH: 255; CK: 105; MMT: 79; CMAS: 49; DAS: 2; NFC: 16/32 (50%) abnormal

Abbreviations: ALT: alanine aminotransferase (U/L), ANA: anti-nuclear antibody, anti MDA5: anti-melanoma differentiation-associated gene 5, anti-transcriptional intermediary factor-(TIF)1γ autoantibodies, AST: aspartate aminotransferase (U/L), Aza: azathioprine, C-HAQ: Childhood Health Assessment Questionnaire, CK: creatine kinase (U/L), CMAS: Childhood Myositis Assessment Scale, CP: cyclosporin, CRP: C-reactive protein, CYC: cyclophosphamide, DAS: Disease Activity Score, EMG: electromyogram, ESR: erythrocyte sedimentation rate (mm/h), GC, glucocorticoids; JDM: juvenile dermatomyositis, JIIM: juvenile idiopathic inflammatory myopathies, JPM: juvenile polymyositis, GC: glucocorticosteroid, HCQ: hydroxychloroquine, IFX: infliximab, IQR: inter-quartile range, IVIG: intravenous immunoglobulin, JDM: juvenile dermatomyositis, kg: kilogram, LDH: Lactate dehydrogenase (mmol/L), MDAAT: Myositis Disease Activity Assessment Tool, MITAX: Myositis Intention to Treat Activity Index, MMT: manual muscle testing, mo: month, MTX: methotrexate, MYOACT: Myositis disease activity assessment, NFC: nailfold capillaroscopy, NR: not reported, PCR: protein-C-reactive (mg/dL); PGA: Physician Global Assessment; PGD: Physician Global Assessment of Disease Damage, QMT: quantitative isometric muscle testing, SRP: signal recognition particle, STIR: short tau-inversion recovery, TAC: tacrolimus, VAS: Visual Analogue Scale, WBC: white blood cell, yrs: years, 3 by 5-Active movement against gravity, 4 by 5-Active movement against gravity and resistance, 5 by 5-Normal power.

**Table 2:** Muscle magnetic resonance protocol details and techniques for imaging acquisition in patients with juvenile idiopathic inflammatory myopathies

Reference	Tesla (T)	Analysed area	Plane	C+	Sequence	Examiners	Acquisition time (min*)	T1 (ms)		T2 (ms)		STIR (ms)			DWI (ms)	
								RT	ET	RT	ET	RT	ET	IT	RT	ET
Hilário et al. [13]	1.5	Th, Glu maximus	Ax, Co	No	T1/T2/STIR	Two radiologists, blinded	NR	500- 640	20	2500	90	1900–2000	20	160	NA	
Kimball et al. [14]	0.5	Th	Ax	No	STIR	One radiologist	NR	NA	NA	NA	NA	1833	30	100	NA	
Huber et al. [15]	1.5	Th, Pe	NR	No	T1/T2/STIR	One radiologist	NR	NR	NR	NR	NR	NR	NR	NR	NA	
Maillard et al. [16]	1.5	Th	Ax	No	T2 relaxation time	One radiologist	NR	NA	NA	NR	NR	NA	NA	NA	NA	
Maillard et al. [17]	1.5	Th	Ax	No	T2 relaxation time	One radiologist	NR	NA	NA	NR	NR	NA	NA	NA	NA	
Sanner et al.[18]	1.0 or 1.5	Th	Ax, Co	No	T1/STIR	Two radiologists, blinded	NR	NR	NR	NR	NR	NR	NR	NR	NA	
Ladd et al. [19]	1.5 or 3.0	Th, Pe	Ax, Co	No	T1/T2/STIR	One radiologist, blinded	NR	400- 500 (1.5T) or 400- 889 (3T)	12–22 (1.5T) or 7–14 (3T)	3500–4500 (1.5T) or 3000–4010 (3T)	76–89 (1.5T) or 58–81 (3T)	4000–5300 (1.5T) or 2930–4720 (3T)	28–37 (1.5T) or 19–44 (3T)	155 (1.5T) or 160- 180 (3T)	NA	
Davis et al. [20]	1.5	Glu muscles, Ad, hamstrings, Qu	Ax	No	T1/T2/STIR	Two radiologists, blinded	9	599	20	3160	94	5900	94	150	NA	
Malattia et al. [21]	1.5	WB	Co	No	STIR	Three radiologists and four paediatric rheumatologists	15–20	NA	NA	NA	NA	2800	165	64	NA	
Thyoka et al. [22]	1.5 or 3.0	Limbs	Ax, Co	No	T1/T2/STIR	Nine radiologists	NR	NR	NR	NR	NR	NR	NR	NR	NA	
Sakurai et al. [23]	1.5 or 3.0	Th, Glu maximus	Ax, Co	No	T1/STIR	Two radiologists, blinded	NR	468- 513	8–11	NA	NA	5840–6700	70–85	150–180	NA	
Mamyrova et al. [24]	NR	Th	Ax	No	T1/STIR	One radiologist, blinded	NR	NR	NR	NA	NA	NR	NR	NR	NA	
Sreelal et al. [25]	3.0	WB	Ax, Co	Yes	T1 (dixon)/ STIR/ DWI	Two radiologists	35–45	3.6	1.3	NA	NA	3460	60	NR	7592	72
Hu et al. [26]	3.0	Th	Ax	No	T2	Two radiologists, blinded	NR	NR	NR	2550–3000	80–87	NA	NA	NA	NA	
Yi et al. [27]	NR	Th, Pe	Co	No	T2	Two radiologists	NR	NA	NA	NR	NR	NA	NA	NA	NA	
Castro et al. [28]	1.5	WB	Co	No	STIR	One radiologist	NR	NA	NA	NA	NA	4900	66	160	NA	

Abbreviations: Ad: adductors, Ax: axial, C+: contrast, Co: coronal, min: minute, DWI: diffusion-weighted imaging, Glu: gluteus, Qu: quadriceps, Th; thighs, ms: millisecond, NA: not applicable, NR: not reported, Pe: pelvis, STIR: short tau inversion recovery, WB: whole-body, RT: repetition time , ET: echo time, IT: inversion time, *rounded to nearest full minute, **thigh region only.

**Table 3:** Muscle magnetic resonance imaging scoring system in juvenile idiopathic inflammatory myopathies

Reference	Abnormality	Muscle evaluation system	Assessed area	Performance of scoring system
Hilário et al. [13]	MO, FO, FR, MA, biochemical shift	Qualitative and quantitative (muscle/fat relationship)	Gluteus maximus, quadriceps, adductors and flexors	Quantitative analysis and muscle weakness: *p* = 0.008; and muscle enzymes: *p* = 0.015 Qualitative analysis and muscle enzymes: *p* = 0.004; and biochemical shift: *p* < 0.05
Kimball et al. [14]	MO, FO, SCO	Qualitative and semiquantitative Likert scale (MO, FO, SCO, SO): 0 (inactive), 1 (mild), 2 (moderate), 3 (severe) and 4 (extremely severe) Likert scale ×body surface (range 0–156)	Thighs and buttocks	Skin STIR MRI with subcutaneous STIR MRI scores (rs = 0.51, *p* = 0.008). Fascial STIR MRI with STIR MRI muscle scores (rs = 0.58, *p* = 0.002). Skin edema on STIR MRI with the clinical assessment of global skin disease activity and aldolase (rs = 0.41 and rs = 0.44, respectively, *p* ≤ 0.04). SCO scores with aldolase (rs = 0.40, *p* = 0.05). MRI MO scores correlated positively with global disease activity scores and LDH (*p* < 0.001).
Huber et al. [15]	MO, MD	Semiquantitative MO: 0 (inactive) to 4 (extremely active) MD: 0 (no damage) to 4 (very severe)	Thighs and lower pelvic musculature	Median MRI MO score at baseline: 2 and follow-up:0 Median MRI muscle damage score at baseline: 0.5 and follow-up: 0.5 CMAS (*p* < 0.004), muscle edema = rs −0.57; muscle damage = rs-0.48.
Maillard et al. [16]	T2 relaxation time measure	Quantitative T2 relaxation time within region of interest (ROI)	Thighs (from the base of the greater trochanters to the level of the superior aspect of the femoral condyles)	MRI scores (slices 4 and 5): (*p* = 0.002). Slices 4 and 5: PGA (*p* = 0.004 and *p* = 0.003), CMAS (*p* = 0.002 and *p* = 0.000), CHAQ (*p* = 0.001 and *p* = 0.000), number of contractures (*p* = 0.005 and *p* = 0.001), MMT neck flexors (*p* = 0.002 and *p* = 0.000), MMT shoulder (*p* = 0.005 and *p* = 0.002), MMT hip abductor (*p* = 0.005 and *p* = 0.009), MMT quadríceps (*p* = 0.001 and *p* = 0.002), MMT hamstrings (*p* = 0.002 and *p* = 0.001) and myometry neck (*p* = 0.017 and *p* = 0.009).
Maillard et al. [17]	T2 relaxation time measure	Quantitative T2 relaxation time within region of interest (ROI)	Thighs (from the base of the greater trochanters to the level of the superior aspect of the femoral condyles)	Active and inactive JDM and controls:No differences: MRI T2-weight relaxation time before exercise, immediately after, and 60 minutes after the exercise (*p* = 0.976 and 0.109)
Sanner et al. [18]	MO, MA, MD, FR, FO, SCO, Cal	Qualitative and semiquantitative SQS: MO and FR − 0 (normal), 1 (fatty streaks), 2 (muscle greater than fat), 3 (muscle equal to fat) and 4 (muscle less than fat) Binary (MO, MA, MD, FR, FO, SCO, Cal)	Thighs	MD:52%; MO: 9%; Median FA score: 0; Median MO score: 0
Ladd et al. [19]	MO, MA, FR, SCO	Qualitative and semiquantitative Ladd system MO, FO and SCO: 0 (no signal abnormality), 1 (1–50% involvement) and 2 (51–100% involvement)	Pelvis (piriformis, superior and inferior gemellus, obturator internus, quadratus femoris, gluteus maximus, medius and minimis), adductors (gracilis, pectineus, adductor magnus, longus, brevis), anterior (rectus femoris, sartorius, tensor fasciae latae, vastus lateralis, intermedius, and medialis), posterior (biceps femoris, semitendinosus, and semimembranosus muscles)	Abnormal reticulated signal intensity in the subcutaneous fat was associated with disease outcome (*p* < 0.02). Progressing to chronic disease was higher for patients with abnormal MRI signal changes in the subcutaneous fat than for patients with normal subcutaneous fat signal (odds ratio, 9.0; 95%CI, 1.5–53.5, sensitivity: 39%, specificity: 91%, negative predictive value:59%, positive predictive value: 82%).
Davis et al. [20]	MO, FO, SCO	Qualitative and semiquantitative SQS: MO − 0 (none), 1 (mild), 2 (moderate) and 3 (severe) FO and SCO − 0 (absent) or 1 (present)	Thighs (gluteal muscles, hamstrings, quadriceps and adductors)	Mean total score for Observer 1: 7.9 and for Observer 2 was 7.5 (mean difference 0.4, 95% limits of agreement 6.8 to 6.0). Mean total scores for Observer 1 were 9.0 for the first reading and 7.9 for the second reading (mean difference 1.0, 95% limits of agreement 2.6 to 4.6).
Malattia et al. [21]	MO, SCO, FO	Qualitative and semiquantitative MO: 0 (none), 1 (mild to moderate) and 2 (high) FO and SCO: 0 (absent) or 1 (increased)	WB	WB-MRI muscle score with clinical disease activity [MMT (rs = −0.84); CMAS (rs = −0.81)] WB-MRI subcutaneous and myofascial scores with PGA of the disease activity, DAS cutaneous component and CHAQ.
Thyoka et al. [22]	MO, FO, SCO, MA, Cal	Qualitative and semiquantitative JIS: Total MO and FO, 0 (normal) to 100 (severe) MO: 0 (non), 2 (mild), 4 (moderate) and 6 (severe) FO: 0 (absent) to 14 (present)	Thighs (adductors, gluteals, hamstrings and quadriceps)	40 axial images: mean JIS for left limb, across the nine raters ranged from 46.8 to 61.0 and the ICC was 0.88 (95% CI: 0.82, 0.92), and for the right limb the mean JIS, across the nine raters, ranged from 47.9 to 61.4 and the ICC was 0.87 (95% CI: 0.81, 0.92). 30 coronal images: mean JIS for the left limb, across the nine raters, ranged from 56.7 to 65.1 and the ICC was 0.90 (95% CI: 0.85 to 0.95), and for the right limb the mean JIS, across the nine raters ranged from 55.7 to 66.8 with an ICC of 0.90 (95% CI: 0.84 to 0.94). The intra-observer reliability and agreement for JIS was also good, with the ICC values between the first and second reads ranging from 0.90 to 0.94.
Sakurai et al. [23]	MO, FO, SCO	Qualitative and semiquantitative Ladd system MO, FO and SCO: 0 (no signal abnormality), 1 (1–50% involvement) and 2 (51–100% involvement)	Thighs (gluteus maximus muscles, adductors and the anterior and posterior compartments)	MRI scores in subcutaneous fat were higher in early than in late-diagnosed JDM (median MRI scores: early, 7, versus late, 0; unpaired two-tailed t-test, *p* = 0.025). Aldolase correlated with MRI score in subcutaneous fat (*p* < 0.0001, ρ = 0.787) and fascia (*p* = 0.013, ρ = 0.574), but not in muscle.
Mamyrova et al. [24]	MO, FR, MA	Qualitative and semiquantitative SQS: MO − 0 (normal), 1 (possible), 2 (definite hyper-intensity < 50% volume), 3 (definite hyper-intensity < 75% volume or severe hyper-intensity < 50%), 4 (diffuse > 75% volume or severe hyper-intensity > 50%) MA − 0 (normal), 1 (possible volume loss), 2 (definite volume loss) and 3 (marked volume loss) FR − 0 (normal), 1 (possible), 2 (definite), 3 ( > 50% and < 75%) and 4 ( > 75%)	Rectus femoris	MRI STIR muscle score (0–4) 0.48 [0–3] MRI T1 fatty change score (0–4) 0.24 [0–2] MRI T1 atrophy score (0–4) 0.38 [0–2] MRI T1 average score (0–4) 0.31 [0–1] Muscle echogenicity on ultrasound correlated with STIR and T1 MRI atrophy scores (rs 0.43). T1 MRI atrophy scores inversely correlated with the median contracted area by ultrasound (rs −0.49). T1 MRI fatty change scores correlated with median rectus femoris resting area (rs 0.57). Average T1 MRI scores correlated with rectus femoris echogenicity (rs 0.55) and difference in rectus femoris area between resting and contracted states (rs 0.62) by ultrasound.
Sreelal et al. [25]	MO, ME, MDF	Qualitative	WB	CK correlated with paraspinal muscles altered signal intensity (*p* = 0.032) and diffusion restriction (*p* = 0.032). Diffusion restriction in the hip muscles (*p* = 0.008) and thigh muscles (*p* = 0.010) with CK. Altered signal intensity (*p* = 0.034) and diffusion restriction in thigh muscles (*p* = 0.001) with LDH. Correlation between strength of the proximal muscles of the lower limb and diffusion restriction in the thigh muscles (*p* = 0.007). Strength in the thigh muscles correlated (*p* = 0.023) with the altered signal intensity. Enhancement in the calf muscles and CK (*p* = 0.040). Diffusion restriction in calf muscles and CK(*p* = 0.043). Strength in the distal muscles of the lower limb and altered signal intensity in the calf muscles. (p 0.002) Strength in the calf muscles and diffusion restriction (*p* = 0.002).
Hu et al. [26]	MO	Quantitative Radiomics score/features from ROI in T2 fat suppression segmented images	Thighs	The nomogram demonstrated great discriminative ability in diagnosing JDM, with an AUC of 0.952 (95% CI, 0.917–0.987) in the training cohort and 0.957 (95% CI, 0.905–1) in the validation cohort. The optimal performance of the combined model used linear discriminant analyses with an AUC of 0.949 (95% CI, 0.912–0.986) and had an apparent improvement compared with the radiomics score model (AUC, 0.912; 95% CI, 0.858–0.967; *p* = 0.02) in the training cohort. Compared with the discriminative ability of biopsy, the combined model developed by the whole patients had a similar performance in JDM discrimination (AUC, 0.952; 95% CI, 0.889–1 vs. AUC, 0.950, *n* = 150; 95% CI, 0.919–0.981, *n* = 72; *p* = 0.95).
Yi et al. [27]	MO, FO, SCO, Cal	Semi-quantitative Likert scale (MO, FO, SCO): 0 (inactive), 1 (mild), 2 (moderate), 3 (severe) and 4 (extremely severe) Binary (Cal)	Pelvis and thighs	Calcinosis and no-calcinosis group: median score: 3 (3 [IQR 1.6, 3.4]; 3 [IQR 2.5, 3.5], respectively) (*p* = 0.39)
Castro et al. [28]	MO	Qualitative Muscle high intensity present or absent	WB	Muscle disease activity was found in 11.8%, confirmed by muscle biopsy. Significant association between WB and abnormal muscle force at the biopsy site (*p* = 0.002). No association between WBMRI and the use of immunosuppression, CK elevation and abnormal capillaroscopy.

Abbreviations: ICC: intra-class correlation coefficients, Cal: calcinosis, DLT: deep learning tool, FO: fascial oedema, FR: fatty replacement, IIM: idiopathic inflammatory myopathy, JIS: JDM imaging score, MA: muscle atrophy, MD: muscle damage, MDF: muscle diffusion restriction, ME: muscle post-contrast enhancement, Myositis Intention to Treat Activity Index (MITAX), MO: muscle oedema, MV: muscle volume, NR: not reported, SCO: subcutaneous oedema, SO: skin oedema, SQS: semi-quantitative score

## Results

**Fig. 1:**
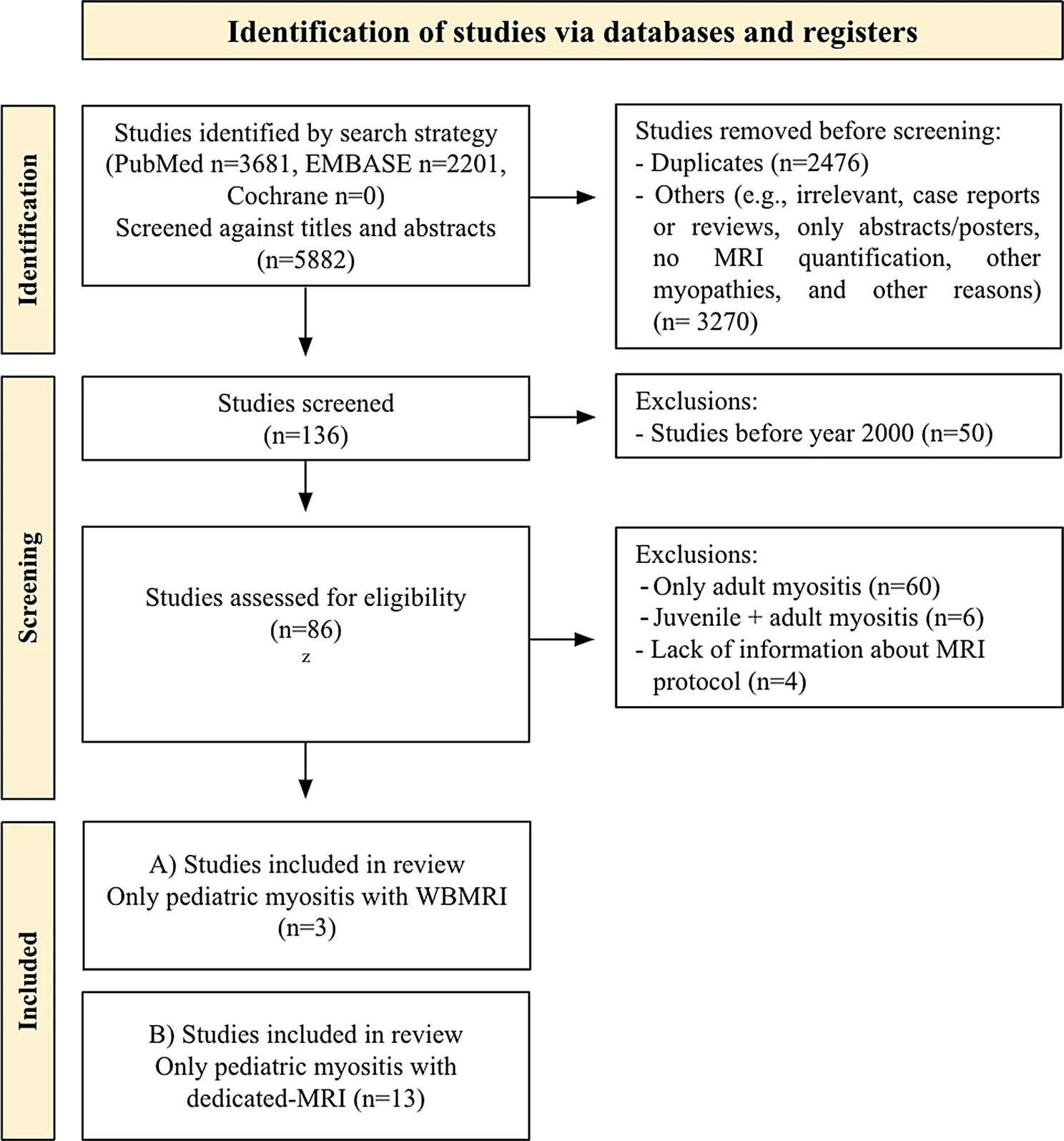
Flowchart of the review for selecting the articles. Ded-BP-MRI: dedicated body-parts magnetic resonance imaging; WBMRI: whole-body magnetic resonance imaging

Our search initially identified 5,882 records, from which 2,476 duplicates were removed. During the screening phase, 3,270 records were excluded for various reasons, including conference abstracts, reviews, decision-analytic models, economic evaluations, or articles addressing etiopathogenic mechanisms of myopathies beyond inflammation (e.g., neuromyopathic etiopathogenesis). Studies in which raw data or additional information were unavailable even after contacting the authors were also excluded, as were duplicate, salami-sliced, or plagiarized publications [12]. The overall workflow is summarized in Fig. 1.

A total of 86 full text articles were reviewed, of which 69 were subsequently excluded based on the inclusion/exclusion criteria of this scoping review. This resulted in three WB-MRI studies [21, 25, 28] and thirteen Ded-BP-MRI studies [13–20, 22–24, 26, 27], including a total of 612 patients with JIIM (595 JDM, 4 JPM, 13 overlap) and 271 healthy controls. Detailed information of the cohort characteristics of primary studies is provided in Table 1, of the MRI techniques and protocols used in primary studies in Table 2, and of MRI scoring systems in Table 3.

## Design of the primary studies

There were eight (50%) cross-sectional [13, 16–18, 21, 22, 24, 28], five (31.2%) retrospective [19, 20, 23, 26, 27] and three (18.8%) prospective studies [14, 15, 25].

## Magnetic resonance protocols

WB-MRI was assessed in three (18.8%) studies [21, 25, 28] and thighs and pelvis Ded-BP-MRI in the other thirteen (81.3%) studies.

A 1.5 Tesla (T) scanner was used in seven (43.7%) studies [13, 15–17, 20, 21, 28], 0.5 T in one (6.2%) [14], and 3.0 T in two (12.5%) [25, 26]. In three (18.7%) studies, 1.5 T or 3.0 T were used [19, 22, 23], and in another one (6.2%), 1.0 T or 1.5 T [18]. Two (12.4%) other studies did not report the scanner used [24, 27].

T1 weighted MRI sequences were performed in ten (62.5%) studies, mainly for assessment of fat infiltration [13, 15, 18–20, 22–25], and T2 or Short Tau Inversion Recovery (STIR) sequences in nine (56.2%) studies [13–17, 19, 20, 22, 26, 27]. Concerning T2 weighted sequences, fat-suppression was used in five (31.2%) studies [15, 19, 22, 26, 27] and relaxation mapping in two (12.4%) studies [16, 17]. STIR fluid-sensitive sequence was carried out in twelve (75%) studies [13–15, 17–23, 28].

Axial and coronal slices were used in six (37.5%) studies [13, 18, 19, 22, 23, 25], axial slices in other six (37.5%) studies [14, 16, 17, 20, 24, 26], and only coronal slices in three (18.7%) studies [21, 27, 28]. Information about MRI data acquisition planes was not reported in one study [15].

With specific regard to WB-MRI, the MRI protocol was performed using a 1.5 T or a 3.0 T scanner with axial and/or coronal STIR sequences [21, 25, 28]. T1 weighted sequences were acquired in only one of three (33.3%) studies [25], with an additional post-contrast T1 weighted acquisition and a diffusion-weighted sequence (DWI). The reported WB-MRI scan times were 35 min − 45 min for those with longer protocols and 15 min − 20 min for those with shorter protocols.

## Reliability of interpretation of measurements

The images were scored by one experienced radiologist in seven (43.7%) studies [14–17, 19, 24, 28], by two radiologists in seven (43.7%) studies [13, 18, 20, 23, 25–27], by three radiologists and four pediatric rheumatologists in one study (6.2%) [21] and by nine radiologists in another study (6.2%) [22].

For WB-MRI, semi-quantitative Quijano-Roy et al. [11] protocol was used in one study for analysis of distribution and extent of disease severity according to muscle edema on STIR images of a 1.5 T WB-MRI. In Malattia et al. [21] study, muscle inflammation was scored using a 0–2 point scale (0 = no muscle signal abnormalities; 1 = mild to moderate signal abnormalities; 2 = high degree of signal abnormalities) considering both the degree of visual signal intensity abnormality and its extent within the entire muscles (more or less than 50% of muscle area affected), which a score of 2 reflecting the presence of areas (even if circumscribed) with high signal intensity and/or signal abnormalities involving more than 50% of the muscle groups. Final score was obtained as an arithmetical sum of all muscular group scores. Perifascicular and subcutaneous tissue inflammation were evaluated using a binary scale (0 = absent, 1 = increase signal intensity) on eight sites (arm, forearm, thigh and lower leg bilaterally), with a total score ranging from 0 to 8. Muscle disease activity was represented by high signal intensity on STIR images in two WB-MRI studies [25, 28].

For Ded-BP-MRI, different methodologies were used in primary studies. STIR images of the thighs were qualitatively graded according to the edema intensity noted in four tissues analyzed (skin, muscle, fascia and fat) using a 0–4 Likert scale: 0 (inactive), 1 (mild), 2 (moderate), 3 (severe) and 4 (extremely severe). In these studies, an edema score was derived by multiplying the intensity of the STIR changes by body surface area, which provided a potential range of 0–156 for these tissues [16]; qualitatively (0 to 1) analyzed images according to muscular and subcutaneous fat signal intensity, biochemical shift, perimuscular edema, muscular atrophy, and muscular fat replacement in gluteus maximus, quadriceps, adductors and flexors in one study [13]. The degree of muscle inflammation was based on a 4-point score (none = 0, mild = 1, moderate = 2 and severe = 3) and presence of soft-tissue oedema (absent = 0, present = 1) and perifascicular oedema (absent = 0, present = 1) were scored in four muscle groups (gluteal muscles, hamstrings, quadriceps and adductors) in other one [20]. Pelvis and thighs were graded according to the intensity of edema on a 0–4 point Likert scale in another [27]. Muscle lesion severity, fascia and subcutaneous fat, analyzed in gluteus maximus muscles, adductors, and the anterior and posterior compartments of the thighs were graded according to Ladd system, where grade 0 = no signal abnormality, grade 1 = 1–50% muscle involvement, and grade 2 = 51–100% muscle involvement [19, 23]. JDM MR Imaging Score (JDM MRI-IS) was calculated ranging from 0 (normal) to 100 (severe) in another study in which muscle, fascia, subcutaneous, and cutaneous layers were scored in both thighs [22]. Edema and calcinosis were scored in all layers. Atrophy, defined as volume loss, was scored in muscle and subcutis. Fatty infiltration was scored in muscle. For all findings, it was used a binary (yes/no) score. Muscle edema and muscle fatty infiltration were scored as: grade 0 = normal, grade 1 = fatty streaks, grade 2 = muscle greater than fat, grade 3 = muscle equal to fat, grade 4 = muscle less than fat. Tumoral, linear, and/or speckled areas with low signal on both T1-weighted spin-echo sequences and STIR sequences were interpreted as fibrosis and/or calcinosis. MRI-detected muscle damage (yes/no) was defined as presenting at least one of the following findings: calcinosis in the muscle or fascia, muscle atrophy, or muscle fatty infiltration [18]. Muscle edema score (range 0–4, where 0 = inactive and 4 = extremely active), and MRI muscle damage score (range 0–4, where 0 = no damage, and 4 = very severe damage) were used for measurements in thighs and pelvis in the study of Huber et al. [15]. T2 relaxation mapping sequence provides data that allow an objective assessment of signal abnormality. In Maillard et al. study [17], regions of interest (ROI) of the thighs were defined where T2 map slice images were obtained. The mathematical means of the readings of these muscles were used to gain an average value of relaxation time for each particular muscle group. Hu et al. [26]. extracted radiomics features from every segmentation of the thigh MRI in T2 fat suppression sequences.

## Measurement analysis

Overall edema and/or inflammation in the skin, subcutaneous tissue and fascia were common in MRI of JDM patients, being associated with further development of calcinosis [14]. MRI was also found to be useful for evaluation of muscle changes in patients with JIIM, including distinguishing normal from pathologic skeletal muscle tissues, defining acute and chronic muscle changes, and detecting and ranking muscle involvement in relation to disease activity status [13, 21]. Although abnormal subcutaneous fat intensity changes appeared to be associated with a more chronic and aggressive disease course, MRI findings could not predict the disease course [19].

STIR MRI was considered a useful adjunct for assessing disease activity and guiding the treatment of JDM [14, 28], correlating with clinical and laboratory disease activity, as well as nailfold capillaroscopy and muscle biopsy features (15). T2 relaxation times were also used as a measure of disease activity [16, 17].

JDM MRI-IS was considered a reliable method for evaluating muscle inflammation in JDM, and routine axial STIR (or T2 weighted fat suppressed) and T1 weighted sequences are recommended [22].

## Quantitative analysis

Quantitative analysis was performed in four (25%) Ded-BP-MRI studies [13, 16, 17, 26].

Hilário et al. [13] evaluated the signal intensity of each muscle group using a region of interest (ROI) of 0.5 cm at the midpoint between the thigh and the lower third of the hip. The relationship between the intensity of the muscle and fat signal was calculated by dividing the signal intensity of the muscle groups by the fat measurements. For each muscle group, the mean of three measurements obtained in the region with the strongest signal of the section was used.

Maillard et al. [16, 17] used T2 relaxation mapping sequences of thigh muscles (anterior, posterior and medial) by defining a ROI in each group and then proceeded applying the mathematical mean ROI scores to obtain an average value of relaxation times. Eight image slices were obtained throughout the patients’ thighs for each scan, with the first slice positioned at the level of the base of the greater trochanters and the final slice positioned at the level of the superior aspect of the femoral condyles. The remaining six slices were automatically positioned equidistantly throughout the thigh.

Hu et al. [26] applied the radiomics score method to obtain quantitative analysis by extracting features from every segmentation of thigh MRI in T2 fat-suppression sequences based on the Py-radiomics library. These authors used a nomogram based on radiomics scores and clinical characteristics to predict the probability of JDM.

## Qualitative and semiquantitative analysis

Qualitative and/or semiquantitative analysis were performed in 12 (75%) out of the 16 articles of this scoping review; three of them the WB-MRI studies [21, 25, 28] and nine Ded-BP-MRI studies [13, 15, 18–20, 22–24, 27].

Overall muscle edema was categorized as 0 (absent) or 1 (present) [13, 18, 25, 28], and as 0 (inactive) to 1 (mild), 2 (moderate), 3 (severe), 4 (extremely severe) [14, 15, 18, 20, 27].

Muscle signal intensity was analyzed in four different ways using categorical intervals. In the first of these scores the muscle signal intensity varies from 0 (0–50%) to 1 (51%-100%) [13, 23]. A second method scores as 0 (no muscle involvement), 1 (1%-25%), 2 (26%-50%), 3 (51%-75%) or 4 (76%-100%) [19]. A third categorical scale utilized 0 (no), 1 (mild to moderate), or 2 (high degree intensity) [21]. The final scale scored as present or absent high intensity [25].

Muscle atrophy was categorized by three different approaches: the first one varied from 0 (1–0.75) to 1 (0.74–0) [13]. A second approach classified muscle atrophy as 0 (absent) or 1 (present) [18]; and the third method was categorized as: 0 (normal), 1 (possible volume loss), 2 (definite volume loss), or 3 (marked volume loss) [24].

Muscle fat replacement was categorized by three different approaches: the first varied from 0 (0–50%) to 1 (51–100%) [14]. A second method was classified as 0 (normal), 1 (fatty streaks), 2 (muscle greater than fat), 3 (muscle equal to fat), or 4 (muscle less than fat) [18]. The third one was categorized as 0 (normal), 1 (possible fat replacement), 2 (definite fat replacement < 50%), 3 (fat replacement 50%-75%), or 4 (fat replacement > 75%) [24].

## WB-MRI studies

Studies utilizing WB-MRI had common goals: evaluating imaging findings compared to clinical and laboratory data and assessing disease activity. Malattia et al. included 41 patients with JDM [21], Sreelal et al. included 21 JDM and nine overlap patients [25] and Castro et al. included 32 JDM and two juvenile polymyositis patients [28].

## Construct validity

### WB-MRI

When WB-MRI was applied, a statistically significant association was observed between WB-MRI findings and abnormal muscle strength at the biopsy site (*p* = 0.002) in Castro et al. study [28]. The results regarding the association with muscle enzymes varied: Castro et al. found no association between WB-MRI and elevated creatine kinase (CK) levels [28], while Sreelal et al. [25] observed a significant positive correlation between MRI edema scores and serum total CK and lactic dehydrogenase (LDH) levels. This positive correlation was associated with altered signal intensity and diffusion restriction in the paraspinal and thigh muscles, as well as with diffusion restriction in the hip and calf muscles [25].

Regarding disease activity, the WB-MRI muscle scores showed moderate to excellent correlations with clinical indicators, such as the Manual Muscle Test (MMT) (rs = −0.84) and the Childhood Muscle Assessment Scale (CMAS) (rs = −0.81). WB-MRI muscle scores also demonstrated moderate to high correlations with the Physician’s Global Assessment of Disease Activity, the Disease Activity Score (DAS) cutaneous component, and the CMAS [21].

## Ded-MRI

Concerning Ded-MRI, positive associations were observed from diagnosis to assessment of clinical and laboratory signs of disease activity. Muscular and subcutaneous fat signal intensity, biochemical shift, perimuscular edema, muscular atrophy, and muscular fat replacement correlated with serum levels of muscle enzymes (*p* = 0.004) and muscle/fat signal intensity correlated with muscle weakness (*p* = 0.008) and muscle enzymes (*p* = 0.015) [14]. Skin global disease activity scores correlated with MRI skin edema scores (*p* = 0.04), and serum aldolase levels correlated with both MRI skin and subcutaneous edema scores (*p* = 0.03 and *p* = 0.05 respectively) [14]. Subcutaneous fat lesions were detected in early diagnosed JDM, suggesting that MRI could be useful in detecting such lesions in order to enable early intervention and prevention of calcinosis [23]. Nevertheless, no association was observed between increased subcutaneous and myofascial edema in MRIs at the time of JDM diagnosis and development of calcinosis in Yi et al. study [27].

Ladd et al. [19] reported abnormal reticulated signal intensity in the subcutaneous fat which was associated with disease outcome (*p* < 0.02). In this study, when controlling for duration of symptoms, the adjusted odds of progressing to chronic disease was higher for patients with abnormal MRI signal changes in the subcutaneous fat than for those with normal subcutaneous fat signal (odds ratio, 9.0; 95% CI, 1.5–53.5). The sensitivity was 39%, with a specificity of 91%, negative predictive value of 59%, and positive predictive value of 82%. However, there was no statistically significant difference in the duration of symptoms between patients who did or did not have abnormal signal intensity in subcutaneous fat (*p* = 0.4742) [19].

Sanner et al. [15] noted significant correlations between CMAS and MRI scores. An increase in T2 relaxation times was found to correlate with an increase in disease activity measured clinically by Physical Global Assessment, CMAS, Childhood Health Assessment Questionnaire Disability Index, and MMT; and with serum levels of CK and LDH [16].

Hu et al. demonstrated an ability of MRI in diagnosing JDM, with an area under the curve (AUC) of 0.952 (95% CI, 0.907- 0.987) in the training cohort and 0.957 (95% CI, 0.905–1) in the validation cohort and investigated the relationship between the radiomics score and clinical variables in children with JDM. Lower muscle strength (OR, 0.75; 95% CI, 0.59–0.90), higher CK level (OR, 1.65; 95% CI, 1.20–2.38), and higher radiomics score (OR, 2.30; 95% CI, 1.63–3.62) were associated with a clinical diagnosis of JDM. The diagnostic value was not weaker than the biopsy (AUC, 0.950; 95% CI, 0.919–0.981, *n* = 150 vs. AUC, 0.952; 95% CI, 0.889–1, *n* = 72; *p* = 0.95) and electromyogram (EMG) (AUC, 0.950; 95% CI, 0.919–0.981 vs. AUC, 0.900; 95% CI, 0.852–0.948; *p* = 0.10) [26].

Mamyrova et al. [24] found that muscle ultrasound (MUS) echogenicity correlated with both STIR and T1 MRI [Spearman correlation coefficient (rs) 0.43], and T1 MRI correlated inversely with rectus femoris contracted area (rs −0.49) on MUS.

## Responsiveness to therapy

Only the study of Castro et al. [28] has assessed the association between MRI findings and therapeutic responses, with no statistically significant association observed between WB-MRI findings and the use of immunosuppression. Concerning disease activity, Malattia et al. [21] reported that the WB-MRI muscle score was significantly higher in active JDM patients compared to both the control subjects (*p* < 0.0001) and inactive patients (*p* = 0.004). In that study, follow-up WB-MRI showed complete resolution of signal abnormalities in nine patients, whereas clinical criteria for remission were met in only five. On the other hand, Maillard et al. [17] found no statistically significant differences in T2-weighted MRI relaxation times when comparing values before exercise, immediately after exercise, and 60 minutes post-exercise in children with active JDM, those with inactive JDM, and healthy controls (*p* = 0.976 - 0.109). Although Thyoka et al. [22] did not have access to clinical data for assessing disease severity, these authors suggested that the JDM MRI-IS is a semi-objective scoring system with potential to serve as a reliable biomarker for both disease severity and response to therapeutic interventions in children with JDM.

## Discussion

This scoping review aimed to identify and summarize existing literature reports for MRI methodologies for the evaluation of JIIM patients and elucidate the possible role of imaging as a diagnostic tool and for measuring disease activity, given that MRI has been widely used by pediatric rheumatologists [29]. Our analysis concluded that out of the 16 included MRI studies, only three used WB-MRI in JIIM, and reported small cohorts of patients and controls using non-standardized methodologies.

Regarding WB-MRI protocols, T1-weighted images acquired on coronal views were the sequences used to measure chronic inflammatory changes, such as atrophy and fat replacement of muscle bellies in one [25] of the three included studies [21, 25, 28]. Coronal STIR was the preferential fluid-sensitive sequence used, employed in all publications, for detecting soft tissue edema (high signal) related to acute or subacute inflammatory findings. Although diffusion sequences (DWI) were added in the WB-MRI protocol of a single included study [25], primarily as a more sensitive sequence to detect edema, additional data are required to estimate the value of DWI in evaluating JIIM. Also, the same study [25] used T1 post-contrast sequences, with no clear evidence of performance improvement.

Concerning Ded-MRI of the lower limbs, the protocols included pelvis and both thighs, were highly heterogeneous with a combination of T1 and fluid-sensitive sequences on coronal and/or axial views, and did not include post-contrast images. T1 images were present in eight studies [13, 15, 18–20, 22–24]. Regarding fluid-sensitive sequences, STIR was applied in nine studies [13–15, 18–20, 22–24], T2 fat-sat was employed in five studies [15, 19, 22, 26, 27]. Additional T2 images were used in studies [13, 16, 17, 20] with relaxation T2-mapping MRI sequences in two of them [15, 17].

The images were scored by one or two experienced radiologists in most studies; however, different scoring systems were used to quantify inflammation using varying scales, ranging from the 0–2, 0–3 to 0–4 scales [15, 18, 20–22, 24], Ladd [19, 23] and Likert [14, 27] scales were applied to validate the JDM imaging score. The use of quantitative scales as an attempt to homogenize imaging reports provided reproducible patterns for potential comparison of clinical parameters, classification, interpretation of disease activity, and progression to chronic changes, despite the heterogeneity of the proposed methods.

In conclusion, although methodologies and objectives differed across the primary studies of this scoping review, the collective findings indicate that MRI is useful to detect muscle, fascial and subcutaneous edema/inflammation and to identify chronic muscle abnormalities. As this study was conducted as a scoping review, rather than as a systematic review, no formal risk-of-bias assessment or quantitative evaluation of heterogeneity was performed, which constitutes a limitation of the reviewing process. Further studies are needed to standardize an optimal MRI protocol in JIIM considering cost-effectiveness, availability and scan time. Moreover, further investigation is required in the future concerning the MRI performance for diagnostic and follow-up of JIIM patients in comparison to other diagnostic methods such as electromyography and muscle biopsy.

## Data Availability

No datasets were generated or analysed during the current study.
